# Postoperative Pain Trajectories in Cardiac Surgery Patients

**DOI:** 10.1155/2012/608359

**Published:** 2012-02-07

**Authors:** C. Richard Chapman, Ruth Zaslansky, Gary W. Donaldson, Amihay Shinfeld

**Affiliations:** ^1^Pain Research Center, Department of Anesthesiology, School of Medicine, University of Utah, 615 Arapeen Drive, Suite 200, Salt Lake City, Utah 84108, USA; ^2^Departments of Anesthesiology and Intensive Care, Friedrich-Schiller University Hospital, 07747 Jena, Germany; ^3^Department of Anesthesiology, Chaim Sheba Medical Center, Sackler School of Medicine, Tel Aviv University, Tel Aviv 69978, Israel; ^4^Department of Cardiac Surgery, Chaim Sheba Medical Center, Sackler School of Medicine, Tel Aviv University, Tel Aviv 69978, Israel

## Abstract

Poorly controlled postoperative pain is a longstanding and costly problem in medicine. The purposes of this study were to characterize the acute pain trajectories over the first four postoperative days in 83 cardiac surgery patients with a mixed effects model of linear growth to determine whether statistically significant individual differences exist in these pain trajectories, and to compare the quality of measurement by trajectory with conventional pain measurement practices. The data conformed to a linear model that provided slope (rate of change) as a basis for comparing patients. Slopes varied significantly across patients, indicating that the direction and rate of change in pain during the first four days of recovery from surgery differed systematically across individuals. Of the 83 patients, 24 had decreasing pain after surgery, 24 had increasing pain, and the remaining 35 had approximately constant levels of pain over the four postoperative days.

## 1. Introduction

Intense pain typically follows surgical procedures, and the control of postoperative pain is still a major challenge [1–9]. In 1998, nearly 32 million Americans underwent 41.5 million surgical procedures, and they spent on the average 5.1 days in hospital [10]. Nearly four decades ago, almost three-fourths of all patients reported moderate-to-severe pain following surgery [11], and one decade ago, this had not changed [12]. More conservatively, an estimated 50–60% of postsurgical patients currently receive inadequate pain control [13]. Failure to control postoperative pain adequately contributes to postoperative morbidity and mortality and drives up the cost of care [14–19]. Pain can persist for a long while after surgery, extending discomfort and slowing rehabilitation [20]. 

Uncontrolled pain following surgery appears to be a risk factor for the development of chronic pain [10, 21, 22]. Thirty-five of 85 postthoracotomy patients still had pain after one year [23]. Eisenberg et al. contacted patients 16 ± 6.3 months after coronary artery bypass grafting [24]. They found that 56% of patients had pain, and 72% of patients reported that the pain interfered with their daily activities.

Good pain control during recovery from surgery requires good pain measurement in the individual patient. Unfortunately, current pain measurement methods suffer from low precision due to unreliability in scores within individuals [25]. Moreover, patients vary systematically and few patients resemble the population average. Postoperative pain normally changes with variation in tissue trauma, it is process dependent, and one of its fundamental features is patterned variation across time [26]. From a process perspective, pain in the recovering surgical patient is a trajectory of finite duration. As a time-limited process, it has such features as a minimum, a maximum, duration, and rate of growth or decline. The common practice of characterizing postoperative pain day by day as a static entity fails to take this into account and thereby loses information that can help define the unique pain management requirements of the individual.

Several statistical approaches under the general rubrics of growth curve modeling [27, 28] multilevel linear models [1] and mixed effects models [29, 30] provide flexible and trenchant techniques for investigating repeated measures [31]. These methods invite evaluation of the trajectory of postoperative pain across days and provide a statistical framework for the evaluation of individual differences in such trajectories. 

Chapman et al. [26] use mixed effects growth curve modeling to examine patterns of postoperative pain in 502 general and orthopedic surgery patients over six days of recovery. Patients provided daily pain reports using a conventional 11-point numerical rating scale. The most parsimonious characterization of an individual trajectory across six measures proved to be a linear fit, termed the acute pain trajectory. With this simple linear model, each patient's trajectory had two key features: (1) the intercept, or initial pain level, and (2) the slope, or rate of pain resolution. The mean intercept (initial pain level) was 5.6, and the mean slope was −.3, denoting that the average patient resolved his or her pain at the rate of about one-third rating unit per day. A mixed effects model analysis revealed significant individual differences in acute pain trajectory slopes. To classify individual patient pain trajectories, the investigators formed a 50% confidence interval around each individual's estimated slope. They classified subjects as probably decreasing in pain over time, probably increasing in pain over time, or staying about the same, according to whether the confidence interval lays strictly above, strictly below, or included zero, respectively. Decomposing the sample into subgroups on the basis of slopes and confidence intervals revealed that 63% of the patients had negative slopes and resolved their pain as expected over six days. However, 25% of the sample had slopes of zero, indicating no pain resolution over the six days while the remaining 12% of patients had positive slopes that revealed steadily increasing pain over six days following surgery. 

The purposes of this paper are to examine the acute pain trajectories over the first four postoperative days in cardiac surgery patients with a mixed effects model of linear growth to determine whether statistically significant individual differences exist in these pain trajectories, and to compare the quality of measurement by trajectory with conventional pain measurement practices.

## 2. Methods and Patients

### 2.1. Modeling and Measuring Individual Responses with Linear Mixed Effects Models

#### 2.1.1. Linear Pain Trajectories

The linear trajectory has two principal features: intercept and slope. The intercept is the level of the pain at the possibly hypothetical time zero assessment point. The second feature, slope, represents rate of change per unit assessment interval. This is the rate at which the pain problem resolves or, in the case of some patients, worsens. Expressed algebraically, the pain score, *Y*, for a given person is a function of two parameters: *Y* = *b*_*o*_ + *b*_1_ *t*, where *b*_*o*_ is the intercept, and *b*_1_ is the slope. The variable *t* represents the point in time at which the measurement occurred.

From a quantitative modeling perspective, a pain trajectory is a type of growth curve, in which growth can be either positive or negative [28, 32–34]. A growth curve represents a pattern of change in measures obtained repeatedly across time (longitudinal data). Each individual in the dataset generates a growth curve, and the features of this curve can serve as a measure for that individual. This advances the concept of individual differences and translates readily to the notion of meaningfully different pain trajectories for different patients. Such measures are less prone to error than individual scores and more informative because they characterize the entire process of change.

#### 2.1.2. Mixed Effects Modeling and Individual Differences

The classical approach to measurement assumes that the individual patient is the only relevant source of information about pain magnitude. It is possible, however, to estimate individual pain levels more precisely by incorporating knowledge about other individuals from the population. Donaldson and Moinpour have presented a general approach to measurement of individual treatment responses in clinical studies that yield repeated assessments on the same individuals [35]. In this method, linear mixed effects models weight the individual estimates of change according to their relative reliability compared with the overall population trends, yielding optimized individual predictions that lie closer to their true values.

Broadly speaking, the individual predictions of change or growth are “measurements” that usually prove superior to classical measures of each patient at each time. Individual trajectories predicted by linear mixed models resemble those for trajectories based exclusively on individual data but are somewhat more precise because they compensate for unreliability inherent in collecting small samples of data from each patient. In the analyses below, we treat the estimated individual trends as constituting the measurements of the underlying postoperative pain processes. The linear mixed model approach is a statistically efficient method for integrating individual and population responses in a single analysis. Similar, though less precise, conclusions would result from obtaining individual trend coefficients for each patient, one at a time, and treating these as measurements of the patients.

### 2.2. Patients

At the Chaim Sheba Medical Center in Tel Aviv, Israel, R. Zaslansky and A. Shinfeld obtained and de-identified pain measures from patients after midsternotomy cardiac surgery. Inclusion criteria included midsternal surgery, extubation, and ability to communicate on the first postoperative day. The Chaim Sheba Medical Center Institutional Review Board approved the project. Patients ranged in age from 23–92 years, with a median of 67. Of the 83 patients, 18 were female. 

Most patients underwent either coronary artery bypass graft surgery or atrial or mitral valve replacement. The primary objective in collecting the data was to assess pain levels and pain medications prior to development of a pain treatment protocol. Patients spent 1 to 2 days in the Cardiac Intensive Care Unit and thereafter transferred to the Cardiac Surgery Ward, where they stayed until discharge. All patients remained in hospital for five or more days after surgery.

### 2.3. Pain Measurement

A single research nurse, trained for the task, collected data from all patients at approximately noon each day, repeating assessment for the first 4 postoperative days. She used a five-category Category Pain Scale (CPS): no pain, mild, moderate, severe, unbearable pain. To evaluate pain during movement, the nurse asked patients to take a deep breath during the first two postoperative days (when pleural drains were still in place) and to cough during the third and fourth postoperative days (after removal of the drains).

Examination of the data on a patient-by-patient basis suggested that patients used the CPS as though the rating categories were equally spaced. An ordinal logistic regression of pain scores on postoperative day provided threshold estimates for the categories, which supported this impression. The distances between the threshold estimates were approximately equal across the range of the CPS. Because the patients used the CPS as though the categories were equally spaced, we treated the CPS ratings as numbers on an interval-level scale. Translated for convenience to numbers on a more familiar 10-point scale, the scores were 0, 2.5, 5, 7.5, and 10. This linear transformation did not distort the data in any way, nor did it alter precision of measurement.

### 2.4. Statistical Approach

We analyzed the data using a linear mixed effects models approach to change trajectories [33, 36]. Based on our previous study [26], we assumed linear time trends, giving rise to Intercept (initial pain at Day 0) and Slope (rate of change in pain per day on study) estimates that can vary across individual patients (the “random effects”) about the Intercept and Slope estimates for the population average trend (the “fixed effects”).

 The mixed effects approach allows each combination of fixed effects to have its own average trajectory, and each patient to have a unique trajectory about the fixed-effects averages. An advantage of mixed-effect models is that, by including both random and fixed effects, they address multiple sources of variation. After allowing for fixed effects, the approach takes into account residual systematic between-subject variation and distinguishes these systematic individual differences from measurement and other error. Another advantage is that such models, unlike conventional longitudinal methods, can accommodate many patterns of systematically missing repeated measures data without bias.

## 3. Results

### 3.1. Group Data

#### 3.1.1. Mean Variation across Days

**Figure 1:**
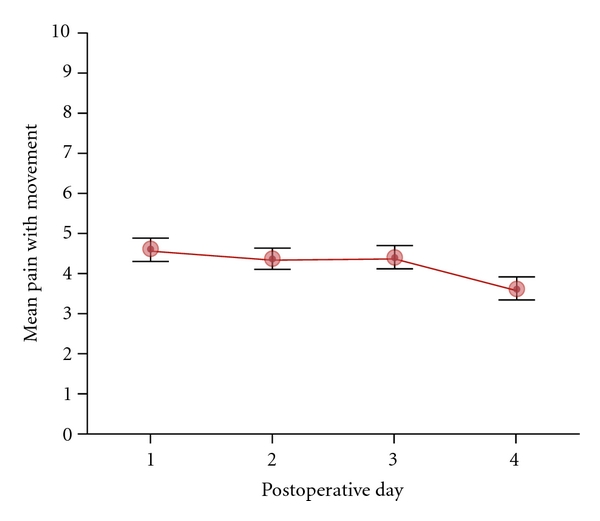
Mean scores (±SEs) for pain with movement across the first four days following sternotomy for 83 patients.

Figure 1 illustrates the means (±SEs) across the four postoperative days for the 83 patients. The means appear to be identical for the first three days and then show a small decline on the fourth day. The SEs indicate that the variability of the scores about the means is large.

### 3.2. Influence of Analgesic Medications

Patients received modest amounts of medication for pain control that included intravenous morphine, oxycodone/acetaminophen tablets, and metamizole. To estimate the potential influence of analgesic medication on pain scores for each postoperative day, we examined intravenous morphine usage on a 24-hour basis, converted 24-hour oral oxycodone intake to its intravenous morphine equivalent, and summed the two to obtain an index of daily opioid intake in intravenous morphine units.

On the first postoperative day, 98% of patients received opioid medication, and the 24-hour mean morphine intake was 7.18 mg. On the second postoperative day, 78% of patients received opioids, and the mean 24-hour intake was 3.54 mg. Seventy-three percent of the patients received opioids on the third postoperative day and 66% on the fourth postoperative day, with a mean intake of 3.21 mg for each day. There was no statistically significant relationship between opioid intake and pain scores on any of the four postoperative days. Analgesic medication use did not distort the pattern of pain reports.

#### 3.2.1. Individual Differences

To evaluate the systematic individual differences in the sample pain trends, we allowed slopes to vary across individuals as a random factor in a mixed effects analysis, [29, 30, 35] that also provided a test of the overall trend (population average slope estimate = −.28 pain points per day, *P* = .018). This analysis, after adjusting for random error, revealed significant (*P* < .0001) variation in the true slopes across individuals (estimated standard deviation = .48). This effect indicates that the patients in our sample do not reflect a homogeneous population sharing a similar trajectory but instead vary systematically about the population average of −.28 with a typical person differing by .48 pain points per day. Thus, a typical patient might have an average pain decline as steep as −.76  (−.28 − .48) points per day, or an *increase* of  .20  (−.28 + .48) points per day. Approximately, 95% of the patients fall within the range defined by an improvement of 1.24 points per day and worsening by  .68 points per day. Variation in observed pain trajectory across the postoperative course includes a pure error component (error variance = 4.34) and also reflects systematic variation due to true differences across individuals. The systematic differences are large; the typical person-to-person difference in slopes (calculated as  .68, or 1.41 times  .48, the standard deviation of the true slopes) is more than twice as large as the average slope difference from zero. The individual differences dominate the average difference.

**Figure 2:**
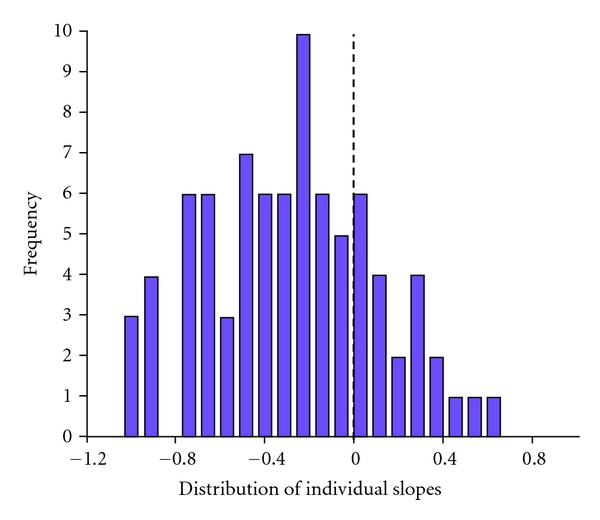
Histogram of the estimated slopes for the 83 individual pain trajectories. The dashed line identifies a slope of zero.

We examined the estimated slopes for important individual differences in pain trajectory.  Figure 2 displays a histogram of the slopes. The dashed line indicates a slope of zero. Negative slopes indicate that the patients gradually reduced their pain over time. A zero slope means that a patient had no pain reduction over the four days while a positive slope indicates that the patient reported a worsening of pain over the four days. The plot reveals that a substantial number of patients have a slope of approximately zero or greater than zero. These observations suggest that nonrandom patterns of postoperative pain may distinguish meaningful patient subgroups.

#### 3.2.2. Grouping Individuals

To classify patients more rigorously, we formed 50% confidence intervals for the mixed model predicted (empirical Bayes maximum likelihood) individual slopes and evaluated whether the intervals contained zero [32]. Patients whose slope confidence intervals were negative at both boundaries qualified as probable “responders.” That is, their pain diminished over the four days following surgery. Those whose confidence intervals were positive at both boundaries qualified as probable “treatment failures.” Their pain grew steadily worse over the four-day postoperative course. The remaining patients (non-responders) had confidence intervals that included zero. Their trajectories were relatively flat over the four postoperative days. The 50% confidence interval classification identified 24 responders, 24 failures, and 35 nonresponders. This grouping reveals that 59% of patients did not demonstrate the expected pattern of gradually reduced pain over days following surgery.

**Figure 3:**
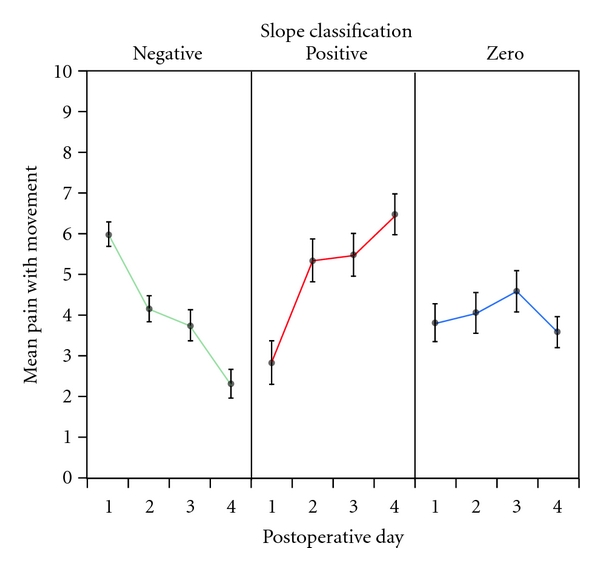
Mean pain with movement scores (±SEs) over postoperative days for patients classified by slopes.

Figure 3 plots the mean pain with movement scores (±SEs) over days, breaking the sample into the three subgroup classifications that emerged from the slope analysis. The means in Figure 1 did not fairly represent the sample, because they averaged over patients with markedly different true responses. In general, opioid consumption was highest in the group of treatment failures, so differences in the aggressiveness of pain management did not cause the variation in the individual slope responses.

### 3.3. Precision of Measurement

The standard error of measurement (SEM) of an assessment gives the typical deviation of an obtained score about the true underlying quantity for that person. Smaller SEMs indicate more precise measurement because typical observations are closer to their true values. The SEM of an assessment is $\sigma \sqrt{1-\rho_{{yy}^{\prime }}}$, where *σ* is the standard deviation of the measurement and *ρ*_*yy*′_ is its reliability. The SEM thus increases with variability among the scores and decreases with the reliability of the assessment.

In our data, independent psychometric criteria suggest that the reliability for the daily pain assessment falls within the range of  .25–.53, values that are quite low by conventional measurement standards. However, an estimated reliability of  .50 for our data is generous. Independent analyses, including an item response theory model that does not assume linearity or interval-level data, confirm that the reliability at the first assessment is about.06, while the reliabilities at the remaining assessments are approximately.4. Under the best scenario, the typical error of measurement is about 1.70 on a 0–10 pain scale for such patients. To have 95% confidence that a single score obtained on a single day contains the true value, one can construct intervals that extend approximately two SEMs on either side of the measured value. For example, the 95% confidence interval for a pain score of 4 is, therefore, 4 ± 3.4: the patient's true score might be as low as 0.6, which does not require intervention, or as high as 7.4, which would merit aggressive medication. A large SEM in pain measurement thus compromises the ability of even the most skilled clinician to control pain.

We evaluated the gain in measurement precision from estimating individual slopes directly versus treating each time point as a separate measurement. Using ordinary least squares regression for a single patient, the estimated error in predicting the expected value of the patient's pain score at time *t*_*j*_ is given by the standard error of estimate for regression: $\hat{\sigma }\sqrt{1/T+{\left(t_{j}-\overset{®}{t}\right)}^{2}/\left(\sum {\left(t_{j}-\overset{®}{t}\right)}^{2}\right)}$, where *T* is the total number of time points for the patient. The average of this prediction error was 1.31, substantially less than the equivalent value of 1.70 for treating the observations as single measures. This gain in precision derived from incorporating a model for all the data for the individual, not just the single observation. The mixed model estimates, which are optimized, obtain somewhat greater gain in precision.

Examination of changes in pain over days revealed more striking gains in precision. The SEM for the difference in pain report between two time points is 41% higher than the SEM for the static assessment. The SEM for the change in measured pain between any two days is 2.40. For the linear model-based approach, the expected error in estimating the change in an individual's expected pain level varies with the temporal distance:.58 for adjacent days, 1.16 for two-day separations, and 1.74 for three-day separations. Thus, for any change, the error is much less than the measurement error of the difference between two assessments considered statically. As in the comparison of individual time points, important gains in precision derive from assuming a model of the postoperative pain process and estimating features of the process rather than individual daily scores. To avoid obscuring this main point, we presented the above comparison based on standard within-person measurement. The mixed model estimate, which is also optimized, actually yields somewhat greater gains in precision than our example indicates.

## 4. Discussion

### 4.1. Individual Variation in Pain Scoring

Although it is sometimes convenient for researchers to treat unexplained variation across patients in pain scores as random variations about a population mean, this does not help clinicians, who must treat patients one at a time and for whom hardly any patient closely resembles the estimated mean of the population. For most experienced clinicians, there is no question that people differ in pain following a common tissue trauma; the only question is whether they differ randomly or in some meaningful way. The commitment to measure pain as an individual clinical outcome signifies acceptance that these outcomes can differ reliably across patients; but how well can we measure these differences that we know are real? 

Pain measurement is the scaling of phenomenal awareness through introspection and report, and this is at most an approximate marker of nociception. Because introspection originated with Fechner and the field of psychophysics [37], most clinicians and researchers intend the numerical pain reports they extract from patients to be indicators of sensory intensity. This is not necessarily the case, however. Clark et al. used cluster analytic techniques [38] to determine that simple, unidimensional rating scales gauge primarily the emotional rather than the sensory dimension of pain. Williams et al. asserted that pain scores reflect the highly individual meaning that each patient imputes to his or her pain to a greater degree than providers normally appreciate [39]. Hodgins pointed out that healthcare professionals lack a common understanding of the meanings behind the scores that pain assessment tools generate, especially in acute care settings [40]. How well simple pain ratings, like the simple scales that clinicians use for pain assessment, reflect nociception, therefore, varies greatly across individuals.

The practical limitations of current pain measurement methods contribute to the high variability in pain ratings, both across and within individuals. One fundamental problem is that not all patients can engage in meaningful introspection and scaling. Another is that the anchors at the upper end of pain scales always have unique meanings for individual patients. For example, “pain as bad as it can be” depends on personal experience and can change with new experience. It may mean a broken leg to one patient, childbirth to another, and a migraine headache to yet another. Consequently, the standard measurement tools scale everyone differently. Experienced clinicians invariably say that a score of “7” from patient X does not mean the same thing as the score of “7” from patient Y, and yet clinicians and researchers treat such scores as though they are in fact the same.

If we treat individual variation in pain scoring as random error, then this variation will cancel out when we average scores over individuals in a large patient sample. Consequently, simple pain ratings often prove informative in clinical trials that examine means. Addressing chronic pain, Rodgers et al. made a cogent point: conventional pain reports work adequately for making group comparisons, but they are not precise enough to indicate, with confidence, how much pain an individual is experiencing [16]. This problem holds in the acute care setting as well, but in this context it stems, not only from the formidable difficulty of quantifying pain in a single scale, but also from an overly narrow characterization of what pain is.

### 4.2. Clinical Implications of a Pain Trajectory Approach to Patient Assessment

Conventional approaches to postoperative pain management focus on controlling immediate pain without regard to controlling the process behind it. In the United States, the Joint Commission for the Accreditation of Health Care Organizations (JCAHO) has introduced standards for the assessment and management of pain in accredited hospitals and other health care settings [41]. JCAHO practice standards hold that providers should intervene when a postoperative pain report reaches a threshold magnitude, for example, three on an 11-point scale. This approach is limiting in two ways. First, it assesses the patient's pain at present, without considering where it has been and where it is going in the future. It is often essential to know whether the pain has been decreasing or increasing. Second, the concept of an interventional threshold presumes minimal pain measurement error. Yet, the pain report that we obtain and record as a score is never more than an approximation of the true pain, and often a very poor approximation, as we have shown. The common clinical dictum, “the pain is what the patient says it is,” may be correct with respect to phenomenology, but not with respect to numbers: that is, it is incorrect from the measurement perspective. All individual scores drawn from subjective report reflect some amount of systematic bias and random error in addition to the true score.

The concept of pain trajectory suggests that the slope of the trajectory, and not the daily score, should be the target of pain control intervention. Our findings indicate that, in at least some postoperative patient populations, significant patterns of individual difference exist in pain trajectories, and clinicians managing postoperative pain should identify such differences as they emerge and treat patients accordingly.

### 4.3. Study Limitations

First, although this study succeeded in demonstrating significant individual differences within a sample of postoperative patients, we did not obtain additional measures that might allow us to characterize the patients within each of the different patterns of postoperative pain resolution, and this reduces the clinical utility of our findings. Numerous studies have attempted to account for postoperative pain intensity and the abnormal persistence of postoperative pain on the basis of patient pathogenic and demographic factors [20, 42, 43], genomics [44], preoperative pain sensitivity [45–51], mood status [52–55], and psychological factors [56–58]. Any of these variables, or combinations of them, could serve as predictors of type of postoperative pain trajectory or rate of postoperative pain resolution. Future studies along these lines are likely to prove fruitful.

Second, in this study, we could follow patients for only four days, and this modest number of repeated measurement occasions constrains measurement precision. In a previous study with over 500 patients, we examined postoperative pain trajectories for six days [26], and in a subsequent smaller study focusing on acute postoperative pain in chronic pain patients undergoing surgery, we tracked postoperative pain trajectories for 14 days [59]. Collectively, these studies demonstrate that linear growth curve modeling is appropriate for postoperative pain trajectories. However, more repeated measures will always yield better measurement precision than fewer repeated measures. The measurement precision for individual estimates depends on the square root of the number of assessments per person. Consider a hypothetical case in which we track our cardiac surgery patients for eight days rather than four. In this case, other things equal, precision with eight assessments stands in the ratio of the inverse square root of 8 to 4, or 71% (29% better) than what we obtained with measures obtained over four days. Thus, investigators using pain trajectories face a tradeoff. Tracking patients over more days increases measurement precision, but it also increases patient attrition rates and study costs. Tracking postoperative pain after discharge is clinically desirable and informative, but this is not normal practice and therefore is labor intensive.

Finally, a linear growth curve model works well for some types of acute pain resolution, especially postoperative pain. However, many forms of acute pain are unsuitable for linear modeling. Some pain states come on slowly, peak, and then resolve gradually. Examples include oral mucositis resulting from anticancer interventions and most headaches. Other acute pains such as menstrual cramping, toothache and the vaso-occlusive pain of sickle cell crisis are irregular and inconsistent across individuals. Pain associated with nephrolithiasis results from a moving and changing noxious stimulus, and pain resolution is unlikely to follow a linear pattern. Therefore, although a linear approach seems to work for postoperative pain, we do not advocate modeling all acute pain with a linear fit. Latent growth curve modeling can use nonlinear trajectories to quantify both systematic change over time and interindividual variability in this change.

## 5. Conclusions

Acute postoperative pain is a dynamic process, and its assessment requires longitudinal measurement. Growth curve modeling of acute pain trajectories reveals that the rate of acute pain resolution is an informative outcome measure that can reveal statistically significant and clinically meaningful individual differences. The common practice of evaluating postoperative pain by using a single measure obtained on the first postoperative day can prove misleading, as a substantial number of patients report low pain initially that steadily worsens as time passes.
